# Clinical Interventions for HIV-Associated Neurocognitive Disorder (HAND): A Systematic Review

**DOI:** 10.1093/geroni/igaa057.3302

**Published:** 2020-12-16

**Authors:** Hannah Mitchell, Erin Robinson, Allison Donehower

**Affiliations:** University of Missouri Columbia, Columbia, Missouri, United States

## Abstract

Despite the widespread use of antiretroviral therapy, HIV-associated neurocognitive disorder (HAND) continues to be one of the most common central nervous system complications of human immunodeficiency virus type 1 (HIV-1). The severity and prevalence of HAND underscores the need for safe, effective therapies to mitigate or eliminate the impacts of the disorder to improve the quality of life of individuals living with HAND. The current study conducted a systematic review of the literature regarding experimental studies of clinical therapeutic interventions for HAND. An electronic search of four databases (PsycINFO, SCOPUS, Ovid MEDLINE, and CINAHL) initially returned 4,280 articles, 31 of which met the inclusion criteria for this study. Articles were selected for inclusion based on several criteria, including the use of a clinical experimental study design and measurement of neuropsychological performance. A large number of studies were excluded due to utilizing observational or cross-sectional designs, relevance, or for otherwise not meeting inclusion criteria. The results of this review revealed 31 articles that investigated both pharmaceutical and cognitive therapies for HAND. Pharmaceutical interventions range from common antiretroviral therapies to novel drug classes with various mechanisms of action. Importantly, this review revealed a number of limitations present in the greater body of HAND research including inconsistencies among methods of diagnosis of HAND and study design, which ultimately make comparisons across studies difficult. This review presents the current evidence that exists regarding therapies for HAND and broadly discusses trends, limitations, and gaps in the literature.

